# Postoperative Combined Modality Treatment in High Risk Resected Locally Advanced Squamous Cell Carcinomas of the Head and Neck (HNSCC)

**DOI:** 10.3389/fonc.2018.00588

**Published:** 2018-12-04

**Authors:** Kedar Kirtane, Cristina P. Rodriguez

**Affiliations:** ^1^Moffitt Cancer Center, Tampa, FL, United States; ^2^Division of Oncology, Department of Medicine, University of Washington, Seattle, WA, United States

**Keywords:** head and neck cancer, squamous cell carcinoma, HPV, head and neck, oropharyngeal carcinoma, nasopharyngeal carcinoma, sinonasal carcinoma, adjuvant therapy

## Abstract

Patients who undergo upfront curative intent resection for locally advanced squamous cell carcinomas and who have adverse pathologic features benefit from adjuvant therapy. Concurrent cisplatin based chemoradiation is an established standard of care endorsed by national guidelines. Controversy now exists on the applicability of this strategy to the good risk human papilloma virus (HPV) related oropharynx cancer (OPC) patient. Ongoing clinical studies are exploring therapeutic de-escalation in the postoperative setting for this distinct patient population. The introduction of immune checkpoint inhibitors to the therapeutic armamentarium for recurrent/metastatic head and neck cancer patients has led to clinical investigation of incorporation of PD-1 inhibition in the postoperative setting.

## Introduction

Therapeutic standards among patients with locally advanced head and neck cancer who have undergone surgical resection have evolved in the past four decades (1). This is largely a result of intense scientific investigation spurred by poor locoregional disease control and patient outcomes. Early studies conducted in the 1970's and 80's focused on defining the role of postoperative radiation therapy and appropriate dosing in patients with high risk features after surgical resection (2, 3). The activity of cytotoxic agents and their radiation sensitizing properties naturally led to studies investigating the efficacy of this combination in both the definitive and adjuvant settings (4, 5).

This review identified landmark prospective clinical trials that provided the foundation for and established current therapeutic standards for postoperative therapy. Pertinent negative trials in the postoperative setting were also included. Ongoing prospective clinical trials in the adjuvant setting were included and cited according to their NCTN identifier. One primary focus is the appropriate postoperative treatment for the prognostically distinct human papilloma virus (HPV) related oropharynx cancer (OPC) with the advent of robotic surgical procedures and the potential for de-escalation in this cohort. Another area of scientific interest involves the incorporation of novel agents to current adjuvant therapy standards, specifically the anti-PD1 inhibitors which are active in the recurrent/metastatic setting (6, 7). The results of these clinical trials are expected to result in refinements in treatment recommendations in the adjuvant setting and improvements in patient outcomes.

## Current Guidelines for Postoperative Treatment

1. Definition of high risk features

The majority of patients with newly diagnosed mucosal squamous cell carcinomas of the head and neck present with locally advanced disease. A proportion of these patients are candidates for surgical resection. In general, oral cavity primaries are approached with surgical resection if feasible, due to the success of surgical reconstruction in this location. Although organ preserving definitive chemoradiation is well established for squamous cell carcinomas originating from the larynx, certain disease characteristics make upfront surgical resection the preferred therapeutic approach (such as laryngeal cartilage invasion). The recognition of adverse pathologic features after surgical resection have been extensively described in literature that dates back to the 1950's, where factors such as advanced T stage, primary site location, nodal disease burden, and surgical margin involvement were associated with high rates of locoregional failure. Subsequent clinical trials in the 1970's explored postoperative radiation in high risk patients, albeit with significant heterogeneity in the definition of high risk. These studies revealed a locoregional and survival advantage to postoperative radiation (2). A combined analysis of two cooperative group studies, Intergroup 0034 [or Radiation Therapy Oncology Group (RTOG) 8503] and RTOG 8,824, sought to define the population at highest risk for poor oncologic outcomes after postoperative therapy. Data from these two early prospective studies confirmed that patients with two or more involved regional lymph nodes, positive surgical resection margins and evidence of extracapsular extension (ECE) were characterized by significantly inferior locoregional control and overall survival rates compared to those without these pathologic characteristics (8, 9). These findings are consistent with a multi-institutional phase III experience which risk stratified patients based on primary site and nodal pathologic features. The study reported inferior locoregional control with postoperative radiation doses < 63 Gy for patients with ECE, and provided further data supporting inferior outcomes in patients with oral cavity primary sites, perineural invasion, ECE, >2 involved LNs (10). These observations provided the foundation for patient selection in the design of clinical trials exploring intensification of therapy in the adjuvant setting.

Apart from pathologic characteristics, timing of radiation therapy appears to influence the outcome of combined modality treatment. Peters et al. (10) and Ang et al. (11) both observed significantly reduced locoregional control in patients with a longer interval between surgery and the initiation of postoperative therapy (10, 11). Similarly, Rosenthal et al. (12) reported a single institution retrospective experience revealing worse locoregional control rates among patients who completed surgery and postoperative radiation over 100 days versus shorter treatment times. This was confirmed by a multivariable analysis that controlled for potential confounders (12). A more contemporary experience has been described by Graboyes et al. (13) who obtained registry data from the National Cancer Database (NCDB) on ~41,000 patients who underwent surgery and postoperative radiation treatment from 2006–2014 (13). Their findings indicate that initiation of postoperative therapy beyond 6 weeks from the date of surgery was associated with worse survival, with survival progressively decreasing with increasing delays. Although it is well recognized that the extent of surgical resection, perioperative complications and patient factors such as insurance status and comorbidity often influence the timing of postoperative treatment, these observations support the timely administration of postoperative treatment. The National Comprehensive Cancer Network (NCCN) recommends the initiation of postoperative therapy ≤ 6 weeks after surgical resection (14).

2. Landmark clinical trials in combined modality postoperative therapy

Recognition of suboptimal outcomes in high risk patients who receive postoperative adjuvant therapy underscored the need for therapeutic intensification in this patient population. Bachaud et al. (15) reported the results of a randomized phase III trial of patients with Stage III–IV resected oral cavity, oropharynx, larynx, or hypopharynx cancers comparing 65–70 Gy postoperative radiation alone to radiation with cisplatin 50 mg/m2 given weekly (15). This phase III trial enrolled 83 patients and revealed superior overall survival and locoregional control in the patients randomized to the cisplatin arm. Similarly, Smid et al. (16) completed a phase III clinical trial examining 56–70 Gy postoperative radiation alone versus radiation with concomitant bleomycin and mitomycin C. The arm with concurrent chemotherapy had superior 2 years locoregional control, disease free, and overall survival (16).

One meta-analyses of chemotherapy in head and neck cancer (MACH-NC) analyzed the results of 63 prospective studies performed from 1965–1993 (17). In the analysis of trials examining postoperative concurrent chemoradiation, chemotherapy administration given during radiation appeared to confer a survival benefit. In contrast, chemotherapy given prior to or after local treatment did not appear to improve survival. These findings paved the way for the design of landmark studies that have established concurrent chemoradiation as a therapeutic standard for high risk resected disease.

The seminal RTOG 9501 trial supported by the Eastern Cooperative Oncology Group (ECOG) R9501 and Southwest Oncology Group (SWOG) 9515 conducted a phase 3 study comparing radiation alone to concurrent chemoradiation with high-dose cisplatin given on days 1, 22, and 43 in patients with squamous-cell carcinoma of the oral cavity, oropharynx, larynx, or hypopharynx who had undergone a complete resection of their disease and had high-risk characteristics (4). The radiation dose in this trial ranged from 60 to 66 Gy in 30–33 fractions over a period of 6–6.6 weeks. After a median follow-up of nearly 46 months, there was a significantly higher rate of locoregional control in the combined modality arm than in the arm that only had postoperative radiotherapy (HR 0.61; *p* = 0.01). The European Organization for Research and Treatment of Cancer (EORTC) trial 22931 conducted a similar study in which patients with stage III/IV squamous cell cancer of the head and neck were randomized to postoperative chemoradiation with high-dose cisplatin versus radiation alone (5).

The maximum radiation dose given was 66 Gy in 33 fractions over a period of 6.5 weeks. After a median follow-up of 5 years, the rate of progression-free survival was significantly higher in the group that received combined modality postoperative therapy (HR 0.75; *p* = 0.04) as was overall survival (HR for death 0.70; *p* = 0.02). Furthermore, the cumulative incidence of local or regional recurrences was significantly lower in the combined-therapy group (*p* = 0.007). It is notable, however, that grade 3 or higher toxicities were more frequently observed in the group that received combined modality postoperative therapy (41% vs. 21 %; *p* = 0.001). The RTOG 9501 intergroup trial and the EORTC trial 22931 played a pivotal role in establishing the current North American guidelines for postoperative combined modality treatment.

It is notable that the two aforementioned studies had varying definitions of high-risk disease. The ECOG R9501/SWOG 9515 study defined high-risk as patients with histologic evidence of invasion of two or more regional lymph nodes, extracapsular extension of nodal disease, and microscopically involved mucosal margins of resection. The EORTC 22931 study defined high-risk disease as positive margins, extracapsular extension of nodal disease, clinical involvement of lymph nodes at levels 4 or 5 for oral cavity or oropharyngeal cancers, perineural disease, and/or vascular embolism. Given the differing definitions, a comparative retrospective subgroup analysis using pooled data from those two trials was published in 2005 (18). The pooled analysis concluded that outcomes for patients with ECE and/or microscopically involved surgical margins was statistically significantly better with combined modality postoperative treatment compared to radiotherapy alone. The subgroup analysis did reveal a trend toward benefit in patients who had stage III–IV disease, perineural infiltration, and/or clinically enlarged level IV–V lymph nodes secondary to tumors arising in the oral cavity or oropharynx, while patients who had two or more involved lymph nodes without ECE did not benefit from chemoradiation. In a long-term follow-up of patients in the RTOG trial with a median follow-up of 9.4 years, patients with microscopically involved resection margins and/or extracapsular spread of disease who received chemoradiation as opposed to radiation alone had lower local-regional failure rates (21% vs. 33%; *p* = 0.02), higher rates of disease-free survival (18% vs. 125; *p* = 0.05), and trends toward an a higher rate of overall survival (27% vs. 20%; *p* = 0.07) (19).

In summary for patients with locally advanced HNSCC who undergo curative intent surgery and have high-risk features (including but not limited to ECE and positive surgical margins), postoperative chemoradiation with cisplatin is the standard of care for those who can tolerate therapy.

3. Clinical investigation with alternative chemotherapy or radiation regimens

Despite the success of the bolus cisplatin and radiation approach, it is well recognized that further optimization of outcomes in high risk populations is needed. Interest in the incorporation of biological therapy into concurrent chemoradiation seemed an attractive approach to intensifying the systemic therapy component, without the excess toxicity of traditional cytotoxic agents. The epidermal growth factor receptor (EGFr) is nearly universally overexpressed among squamous cell malignancies, and inhibitors appeared to have preclinical synergy with radiation therapy. In the definitive treatment of locally advanced oropharynx, larynx, and hypopharynx cancers, the combination of cetuximab with radiation therapy resulted in superior overall survival, progression free survival, and locoregional control (20).

Harrington et al. (21) reported the results of a multicenter phase III clinical trial which randomized 688 patients with Stage II-IVA resected squamous cell carcinomas of the head and neck to cisplatin based concurrent chemoradiation (to 66 Gy) with either lapatinib, an oral EGFr inhibitor, or placebo (21). Patients continued lapatinib or placebo for a maintenance period lasting 12 months after completion of chemoradiation. This study was terminated early due to findings that there was no difference in the primary endpoint of disease-free survival. The randomized phase II RTOG 0234 trial investigated postoperative concurrent chemoradiation (60 Gy) with cetuximab and the addition of either cisplatin or docetaxel in 238 patients with high risk resected disease defined as positive margins, ECE, or two or more nodal metastases. Although not designed to compare both arms, the 2 years disease-free survival was encouraging in the non-cisplatin containing docetaxel/cetuximab arm, leading to the design of the phase III RTOG 1216 trial (22). It is of note that trials in the definitive setting for locally advanced HNSCC incorporating EGFr inhibition (cetuximab, erlotinib, panitumumab) with chemoradiation have failed to show an advantage in progression-free survival or overall survival over chemoradiation alone (23–25).

Similarly, interest in variations of platinum administration is of particular clinical significance as standard bolus cisplatin based chemoradiation results in significant high grade toxicity. For example, the EORTC 22931 study reported that only 61% of the study patients were able to complete all three planned cisplatin doses in patients randomized to chemoradiation. Argiris et al. (26) conducted a phase III clinical trial comparing postoperative radiation (at least 60 Gy) alone to radiation with concurrent weekly carboplatin 100 mg/m2 (26). This study was terminated early due to poor accrual, and analysis of the 72 patients randomized showed no difference in 5 years disease-free survival and overall survival in the two arms. Noronha et al. (27) reported a phase III trial conducted in India of 300 patients randomized to bolus 100 mg/m2 cisplatin or weekly cisplatin given at 30 mg/m2 given concurrently with radiation (27). The overwhelming majority (90%) of patients enrolled were treated in the postoperative setting for oral cavity primary sites (87%) with the most common high risk feature being ECE. Inferior locoregional control was observed in the arm receiving weekly cisplatin. There was a trend toward superior overall and progression free survival (PFS )in the arm receiving the bolus cisplatin group. It is also notable that this study was criticized for its use of 30 mg/m2 dosing rather than the 40 mg/m2 that is more commonly used in practice. The Japan Clinical Oncology Group is currently conducting a randomized phase II/III study (JCOG1008) of weekly (40 mg/m2) vs. bolus (100 mg/m2) cisplatin given concurrent with postoperative radiation in patients with resected high risk HNSCCs (28). Lastly, one meta-analysis of 52 studies found that there was no difference in OS or response rate between low-dose weekly and high-dose three-weekly cisplatin regimens (29). However, given that this has not been prospectively studied in a randomized clinical trial and the Japanese study results are still pending, the standard of care still remains high-dose cisplatin.

Finally, studies of alternative fractionation in locally advanced HNSCC have failed to show a survival benefit (30, 31). A Phase III study comparing accelerated versus fractionated postoperative radiotherapy for advanced head and neck cancer did show a trend for improved locoregional control for patients who had a delay in starting radiation, but otherwise no significant differences were seen between the control and experimental arms (30). One meta-analysis of six trials involving more than 900 patients with locally advanced HNSCC found that accelerated radiation therapy did not improve loco-regional control, progression-free survival, or overall survival (32). In fact, the meta-analysis found that accelerated radiation therapy schedules were associated with higher rates of acute mucositis.

## Ongoing Clinical Investigation

1. HPV-related oropharynx cancer

Increasing recognition of the HPV-related oropharynx squamous cell carcinoma subset has had a tremendous impact on the prospective evaluation of HNSCC. This distinct entity, which carries a superior prognosis both in the locally advanced and the metastatic setting, has led to the design of HPV OPC specific clinical trials and has necessitated stratification for HPV status when studied with non HPV related HNSCC (33). Given the nontrivial toxicities of postoperative chemoradiation, a natural research question in this population is whether de-intensification of therapy would result in similar or better oncologic and quality of life outcomes. One particular controversy is the significance of ECE in patients who have undergone resection for HPV related OPC, since the current therapeutic standard established by RTOG 9501 and EORTC 2291 was studied prior to the recognition of the HPV related OPC as a separate entity. Provocative reports from various single institution studies (34, 35) suggest that in contrast to the previously described experience prior to the HPV era, ECE does not appear to influence outcomes in HPV related OPC patients treated with upfront transoral robotic surgery.

Prospective data is expected from ECOG 3311, a recently completed, randomized, prospective phase II clinical trial for patients with advanced stage HPV associated oropharyngeal squamous cell cancer who have undergone transoral surgery and neck dissection (NCT01898494). In this trial, patients with high-risk features (i.e., positive margins, ECE, or ≥5 greater metastatic lymph nodes) were assigned to receive standard of care adjuvant chemoradiation therapy. Patients with no high-risk features were assigned to observation (i.e., no adjuvant therapy); those with intermediate-risk features (close margins, perineural invasion, lymphovascular invasion, or 2–4 metastatic lymph nodes) were randomized between standard- (60 Gy/30 fractions) and reduced-dose radiation (50 Gy/25 fractions). The trial's primary objective will be to evaluate 2 years progression free survival (PFS) in HPV-positive HNSCC patients treated with low-dose radiation therapy, while its secondary end points will be early/late toxicities, quality of life, and swallowing function.

Two other large postoperative de-escalation Phase III trials are ongoing. PATHOS (NCT02215265) is a multi-institutional randomized trial conducted in the United Kingdom similar to ECOG 3311 that will risk stratify HPV related p16+ HNSCC into low, intermediate or high risk groups. Patients with intermediate risk will be randomized to standard or deescalated postoperative radiation. High risk patients will be randomized to postoperative radiation to 60 Gy or postoperative radiation with weekly cisplatin chemotherapy. ADEPT NCT01687413 trial is a study of postoperative adjuvant therapy de-intensification for HPV-related, p16+ oropharynx cancers. In this trial, HPV-related oropharyngeal cancer patients who have undergone surgery and neck dissection and have been found to have high-risk features are randomized to standard-of-care adjuvant chemoradiation versus radiation alone.

In addition, smaller phase II trials are being conducted in North America. The Sinai Robotic Surgery Trial (SIRS - NCT02072148) is a single institution trial which will risk stratify patients to low, intermediate or high risk. Patients with intermediate risk will receive 50 Gy postoperatively, and those with high risk treated with 60 Gy with weekly cisplatin chemotherapy. The Mayo Clinic NCT01932697 is conducting a phase II trial wherein patients with high risk features after resection will be treated with altered fractionation with concurrent docetaxel. The University of Pennsylvania (NCT02159703) has an ongoing clinical trial involving adjuvant radiation to the regional lymph nodes only (sparing the primary sites) in patients with low volume T disease, negative margins and pathologic nodal involvement. Lastly, a large German multi-institutional Phase I study, DELPHI (NCT03396718), is ongoing and is examining deescalated radiation doses in patients with low or intermediate risk pathologic features after resection.

It is notable that in the definitive setting, de-intensification studies for HPV-related oropharyngeal cancers have preliminarily shown negative results. RTOG 1016 (NCT01302834) is an ongoing trial comparing radiation therapy with cisplatin or cetuximab in patients with p16 positive oropharyngeal cancers. For now, the question of whether patients with locally advanced HNSCC which are HPV-positive require the same intensity of adjuvant therapy as those which are HPV-negative remains unanswered. Given the preliminary negative studies reported in the definitive setting, it is important to note that de-escalation still has high-risk and should be tested only within the context of a clinical trial. Table 1 summarizes the selected de-escalation studies of adjuvant therapies in HPV-positive head and neck cancers.

**Table 1:** Summary of selected HPV de-escalation studies in the adjuvant setting.

**NCT Identifier**	**Phase**	**Intervention**
ECOG 3311—NCT0198494	II	Pathologic risk stratification after transoral surgery. Low-risk patients are observed; intermediate-risk patients are randomized between 50 and 60 Gy of radiation; high-risk patients receive 66 Gy with weekly cisplatin
PATHOS—NCT02215265	III	Pathologic risk stratification after transoral surgery and neck dissection. Low-risk patients are observed; intermediate-risk patients are randomized between 50 and 60 Gy; high-risk patients are randomized between 60 Gy +/– concurrent cisplatin
ADEPT—NCT01687413	III	Patients with extracapsular extension are randomized to 60 Gy of radiation +/– concurrent cisplatin
SIRS—NCT02072148	II	Pathologic risk stratification after transoral surgery. Low-risk patients are observed; intermediate-risk patients will receive 50 Gy radiation; high-risk patients will receive 60 Gy of radiation with concurrent cisplatin
Mayo—NCT01932697	II	Patients found to have pathologic high-risk after resection will be treated with altered fractionation with concurrent docetaxel
Penn—NCT02159703	II	Adjuvant radiation to regional lymph nodes in patients with low volume T disease, negative margins, and pathologic nodal involvement
DELPHII—NCT03396718	I	Examines deescalated radiation doses in patients with low or intermediate risk pathologic features after resection

2. Immunotherapy and other therapeutic strategies in the perioperative setting

Nivolumab and pembrolizumab, two monoclonal antibodies that inhibit PD-1, were approved in 2016 for recurrent/metastatic squamous cell carcinomas of the head and neck previously treated with cisplatin chemotherapy. The encouraging activity of these agents in the metastatic setting gives merit to investigation in the curative intent setting. Preclinical data suggests synergy between the anti-PD-1 inhibitors and radiation therapy, making this approach an attractive one in the high risk postoperative setting (36).

The NRG has recently completed HN003 (NCT02775812), a phase I experience in high risk resected HPV related squamous cell carcinomas (defined as positive margin and/or ECE), wherein pembrolizumab 200 mg IV every 3 weeks administered with postoperative radiation and weekly cisplatin chemotherapy. The results of this study are expected to provide a basis for future comparison of this regimen to the current therapeutic standard. Another strategy under study is the administration of the anti-PD1 agents in both the neoadjuvant and adjuvant setting. Wise-Draper et al. (37) recently reported the preliminary safety data of an ongoing phase II trial (NCT02641093) wherein patients with locally advanced resectable HNSCC received one dose of pembrolizumab 1–3 weeks prior to surgery (37). All patients received postoperative radiation to 60 Gy with concurrent pembrolizumab 200 mg IV every 3 weeks × 6 doses, or the same regimen with concurrent weekly cisplatin (40 mg/m2) in patients with high risk features. At the time of reporting, 28 patients had been enrolled, and 9 of 19 evaluable patients had evidence of pathological response on examination of the surgical specimen. No dose limiting toxicities were observed. Another phase II trial (NCT02296684) is ongoing with a similar neoadjuvant/adjuvant pembrolizumab design, but with bolus cisplatin 100 mg/m2 given with postoperative radiation in high risk patients. An early report on the first 21 patients enrolled showed encouraging tolerability of the pre-surgical pembrolizumab dose with no patients experiencing delays in surgery or unexpected complications (38). Furthermore, 43% of patients showed a pathologic response on the surgical specimen. Table 2 summarizes selected immunotherapy trials that are ongoing.

**Table 2:** Summary of selected immunotherapy trials.

**NCT Identifier**	**Target**	**Trial**
NCT02841748	PD-1	Randomized, Double-Blind Phase II Study of Adjuvant Pembrolizumab Vs. Placebo in Head and Neck Cancers at High Risk for Recurrence
NCT02296684	PD-1	Immunotherapy with MK-3475 in Surgically Resectable Head and Neck Squamous Cell Carcinoma
NCT02775812	PD-1	Cisplatin, Intensity-Modulated Radiation Therapy, and Pembrolizumab in Treating Patients with Stage III–IV Head and Neck Squamous Cell Carcinoma
NCT02641093	PD-1	Phase II Trial of Adjuvant Cisplatin and Radiation with Pembrolizumab in Resected Head and Neck Squamous Cell Carcinoma
NCT03325465	PD-1; IDO1	Neoadjuvant Pembrolizumab + Epacadostat Prior to Curative Surgical Care for Squamous Cell Carcinoma of the Head and Neck
NCT02741570	PD-1; CTLA-4	Study of Nivolumab in Combination with Ipilimumab Compared to the Standard of Care (EXTREME Study Regimen) as First Line Treatment in Patients with Recurrent or Metastatic Squamous Cell Carcinoma of the Head and Neck

*PD-1, Programmed cell death protein 1; IDO1, Indoleamine 2,3-dioxygenase-1; CTLA-4, Cytotoxic T-lymphocyte-associated protein 4*.

Alongside immunotherapy, a number of other therapeutic strategies are currently under active investigation. The ECOG-ACRIN cancer research group is studying the use of radiation therapy with or without cisplatin in treating patients with p16 negative stage III-IVa HNSCC who have undergone surgery (NCT02734537). This phase II trial mandates central determination of tumor p53 status, and is anticipated to provide valuable information regarding oncologic outcomes based on p53 aberrations, potentially paving the way for genomically driven adjuvant therapy recommendations. Furthermore, translational research is pushing the field toward the development of novel therapeutic strategies, with synthetic lethality proving to be an encouraging avenue for therapy. Increasing preclinical evidence points to the reliance of p53 deficient cells on wee-1, a G2/M checkpoint regulator, to affect DNA repair after exposure to cytotoxic agents. The wee-1 inhibitor—AZD1775—in combination with neoadjuvant weekly docetaxel and cisplatin before definitive therapy in HNSCC had promising findings that may be translated into an innovative therapeutic approach (39). An ongoing trial (NCT03028766) will seek to combine the wee-1 inhibitor with cisplatin and radiotherapy after surgery in patients with HNSCC.

## Summary

While concurrent cisplatin based chemoradiation continues to be the standard of care for postoperative management of high risk resected HNSCC, the field is rapidly changing. Improving radiation techniques, checkpoint inhibitors, and novel therapeutic strategies, along with the recognition of HPV status as an important prognostic indicator, may help to increase the probability of cure in patients with advanced head and neck cancers. Ongoing clinical trials will hopefully be able to answer how to rationally combine effective novel therapies in the postoperative setting.

## Author Contributions

All authors listed have made a substantial, direct and intellectual contribution to the work, and approved it for publication.

### Conflict of Interest Statement

The authors declare that the research was conducted in the absence of any commercial or financial relationships that could be construed as a potential conflict of interest.
